# Does Foreign-Accented Speech Affect Credibility? Evidence from the Illusory-Truth Paradigm

**DOI:** 10.5334/joc.353

**Published:** 2024-02-19

**Authors:** Anna Lorenzoni, Rita Faccio, Eduardo Navarrete

**Affiliations:** 1 Dipartimento di Psicologia dello Sviluppo e della Socializzazione, Università degli Studi di Padova, Padova, 35131, Italy

**Keywords:** Foreign-accented speech, Credibility, Fluency processing, Illusory-truth effect, Intelligibility, Foreignness, Out-group stereotype

## Abstract

In a pioneering study, Lev-Ari and Keysar (2010) observed that unknown statements are judged less credible when uttered with foreign accent compared to native accent. This finding was interpreted in terms of processing fluency; when intelligibility is reduced, the credibility of the message decreases. Here, we use the illusory truth paradigm to explore how accent affects credibility. In a between-participant design, participants were exposed to unknown statements uttered by native-accented or foreign-accented speakers. After a distractor task, the same statements were presented with new statements, and participants assessed their truthfulness. Truthfulness ratings were higher for repeated statements than for new statements, replicating the illusory truth effect. Contrary to the processing fluency hypothesis, the effect was similar in both the foreign-accented and native-accented speech groups. A new group of participants rated the speakers’ voices on various social traits. A negative bias against foreign speakers was observed. However, this negative-bias did not affect truth ratings.The impact of foreign-accented speech on message credibility is discussed in the context of two factors, processing fluency and out-group stereotype activation.

## Introduction

Several characteristics of the speech signal may determine how listeners interpret a spoken message. For instance, indexical properties of the voice, such as pitch or volume, can influence the way a sentence like “I have a large tattoo on my back” is interpreted, depending on whether the voice is associated with a child or an adult (Van Berkum et al., 2008). Another characteristic of speech is the accent, which is defined as the idiosyncratic way in which each speaker pronounces words and sentences of a language. A topic that has received significant attention in the cognitive and social sciences is whether the accent used to convey a spoken message affects its interpretation. Here, we explore whether accent modulates message credibility by contrasting foreign-accented and native-accented speech.

The accent of a speaker is shaped by their linguistic and cultural heritage. Listeners naturally and effortlessly draw upon this information, forming distinct personal impressions of speakers based on their accent (Cargile & Giles, 1997; Cargile et al., 1994; Ryan, 1983). It has been suggested that a negative bias exists in adult listeners, as they typically judge speakers with a foreign-accent as less trustworthy, less educated, less intelligent, and less competent than native-accented speakers (Dewaele & McCloskey, 2015; Fraser & Kelly, 2012; Fuertes et al., 2012; Giles & Watson, 2013; Gluszek & Dovidio, 2010). This negative bias may have two origins (Foucart et al., 2020). One origin is socially-driven and initiates with the automatic categorization of a foreign-accented speaker as an out-group individual (Baus et al., 2017; Baus et al., 2021; Champoux-Larsson et al., 2022; Kinzler, Dupoux & Spelke, 2010; Pietraszewski & Schwartz, 2014a; Lorenzoni et al., 2022a). The sensitivity toward accents is evident from childhood (Kinzler et al., 2007) and it remains stable in adulthood (Pietraszewski & Schwartz, 2014b). Importantly, the automatic categorization linked to foreign accents seems to trigger stereotypes and stigmas associated with the respective foreign groups (Ryan, 1983; Wated & Sanchez, 2006). The activation of these stereotypes may affect the trustworthiness of the message, influencing message credibility (Giles & Watson 2013; Ryan, 1983; Stevenage, Clarke & McNeill, 2012).

A second origin of the negative bias refers to the fact that foreign-accented speech deviates from the standard pronunciation of the listener. Consequently, foreign-accented speech is often perceived as less fluent than native-accented speech and can be more challenging to understand. Several studies support the claim that processing difficulty is inversely related to the truth-value assigned to the message (i.e., the processing fluency hypothesis, Oppenheimer, 2008). Cognitive fluency refers to the ease and speed with which people process information, and it is related to the concept of mental (cognitive) effort required to complete a task. The amount of mental effort involved in solving a task determines not only the outcome but also how people perceive the information regarding the task. Those tasks that require less mental effort can lead to greater confidence during task resolution and a more positive overall experience. That is, the information that is presented under these circumstances is easy to process and understand and, at the same time, it tends to be perceived as more familiar and trustworthy (e.g., Oppenheimer, 2008). Contrarily, under circumstances of more cognitive mental effort, information is more difficult to process and understand. This information is perceived as unfamiliar and untrustworthy, leading to a negative experience. In a pioneering study, Reber and Schwarz (1999) manipulated the colour font of several statements and the background colour of the screen on which they were presented. In doing so, some statements were easier to read than others. Statements were judged as truer when they were presented in easier-to-read colour contrasts.

In relation to spoken language, foreign-accented speech diverges from standard (native) speech; therefore, it is perceived as less fluent. The inherent difficulty in processing foreign accent may negatively affect the credibility of the message (Dragojevic et al., 2017; Schwarz, 2004; Formanowicz & Suitner, 2020). Lev-Ari and Keysar (2010) tested for the first time the hypothesis of ‘easier to process-easier to believe’ in the context of message credibility. In their study, native English speakers were exposed auditorily to 45 trivia statements about facts of world knowledge that were mostly unknown to the population (e.g., ‘Ants don’t sleep’) and were required to judge their veracity on a 14 cm line. Three native English speakers, three non-native speakers of English with a mild accent, and three with a heavy accent recorded the statements. In two experiments, trivia statements were judged as less true when spoken by a non-native than a native speaker (see also Hanzlikova & Skarnitzl, 2017). Lev-Ari and Keysar rejected any role of stereotypes of prejudice against foreigners because their participants were informed that the speakers they heard were merely reciting statements provided by a native speaker. Instead, they interpreted the results in terms of processing fluency: participants perceive the statements as less truthful because they perceive them as more difficult to understand.

In a recent follow-up study, Boduch-Grabka & Lev-Ari (2021) replicated the observation of Lev-Ari and Keysar that statements are judged as less credible when produced with a foreign-accented speech than native-accented speech. Critically, this difference was reduced when participants were previously exposed to the same foreign-accented speech before the judgment test (in their experiment, Polish-accented English). The authors concluded that the exposure to a foreign accent increases speech processing for this accent through a mechanism of adaptation. Congruent with this conclusion, Clarke and Garrett (2004) showed that, on a cross-modal word verification task, exposure to less than 1 minute of speech by a foreign-accented talker is sufficient for listeners to overcome the initial decrease in processing speed for foreign-accented versus native-accented speech. More recently, Rovetti, Sumantry, and Russo (2023) found that a brief exposure (less than 2 minutes) to foreign-accented speech increases intelligibility and reduces processing effort.

However, it must be said that other researchers failed to find an effect of foreign-accented speech on message credibility. For example, Souza and Markman (2013) adopted Lev-Ari and Keysar’s (2010) design and manipulated the speech signal by adding white or babble background noise to the statements. Forty-eight statements with three levels of noise were presented in two conditions (i.e., white noise vs. babble noise). Participants were asked to rate the truthfulness of the statements on a 10-point Likert scale. The results did not reveal an effect of noise level on the truthfulness ratings. In a second experiment, Souza and Markman (2013) attempted to directly replicate Lev-Ari and Keysar’s (2010) study and the same statements were used, but this time recorded by native English speakers and foreign-accented speakers (Brazilian-Portuguese and Korean). The results of this second experiment did not replicate the findings of Lev-Ari and Keysar (2010) as participants did not differ in the credibility ratings they attributed to the statements recited in the native or foreign accents (see also Foucart & Hartsuiker, 2021; Podlipský, Šimáčková & Petráž, 2016; Foucart, Santamaría-García & Hartsuiker, 2019 for a partial effect of accent on trust). Again, Stocker (2017) attempted to replicate the findings of Lev-Ari and Keysar (2010) in the Swiss context, using different types of accents (Italian, English, Swiss-German and French). French and Swiss-German participants completed the experiment. The results of statement ratings did not indicate any influence of foreign accent on credibility, and the response patterns did not differ systematically between the French and Swiss-German accent.

In summary, existing research on the impact of accent on message credibility has yielded inconsistent results, with some studies reporting an influence and others not. We aim to contribute insights to this ongoing debate. Past research proposes that two factor could influence the credibility of messages delivered in foreign-accented speech compared to native-accented speech. One factor relates to the categorization of the foreign speaker as an out-group individual, which may, in turn, activate stereotypes associated with that group. The second factor is linked to the perception that foreign-accented speech is less fluent than native-accented speech, deviating from the standard pronunciation expected by the listener. Here, we implemented a design that attempts to mitigate the influence of the social/stereotype activation factor. However, it is crucial to note that the two factors are intricately connected within the oral message; foreign-accented speech is characterized as less fluent and is automatically classified as belonging to an out-group individual. Our study provides a conceptual replication (Agnoli et al., 2021) of the impact of accent on message credibility by studying some critical features in relation to previous research. Below we detail these features.

First, several studies have used a within-participant design in which participants were exposed to both native and foreign-accented speech (e.g., Lev-Ari & Keysar, 2010; Boduch-Grabka & Lev-Ari, 2021; Hanzlikova & Skarnitzl, 2017). This could increase the categorization of the speakers as in-group or out-group individuals (Tajfel et al., 1971). Indeed, one could argue that the exposure to both in-group and out-group creates an immediate categorization, because the participant is forced to compare the two groups with each other. In other words, presenting both accents could prompt participants to activate the stereotypes associated with the corresponding groups. As mentioned above, the activation of stereotypes may influence message credibility (Giles & Watson, 2013; Ryan, 1983; Stevenage, Clarke & McNeill, 2012). We adopted a different procedure and used a between-participant design in which two groups of native Italian speakers were instructed to rate the credibility of a series of statements. One group was tested with statements produced by native speakers of Italian, while the other group of participants was tested with statements produced by foreign-accented speakers of Italian. This between-participant design should reduce the social categorization of the speakers.

Second, in our study we employed an implicit task directly linked to cognitive fluency. A critical key in cognitive fluency is repetition, and it is well-demonstrated in the literature that repetition increases fluency (e.g., DiFonzo et al., 2016; Hassan & Barber, 2021; see also Schnuerch, Nadarevic & Rouder, 2021). Critically for our purposes here, repeated information is perceived as more truthful than new information, as evidenced by the illusory truth effect (Hasher, Goldstein, & Toppino, 1977). There are different paradigms on the illusory truth effect; in one of the most popular, participants judge the truthfulness of a series of unknown statements (e.g., ‘Ants don’t sleep’). Half of these statements are presented for the first time. The other half of the statements have been previously encountered in an encoding phase, in which participants are instructed to read or to perform a different distractor task. Statements that have been presented twice tend to be considered more truthful (see Brashier and Marsh, 2020 for a recent review) in relation to statements that are seen for the first time. Here we adopted this paradigm to test the interaction between processing fluency and foreign-accented speech on message credibility.

To the best of our knowledge, the study by Frances, Costa, and Baus (2018) is the only one that has used the illusory truth paradigm to explore the role of accent in credibility. In Experiment 3 of this study, native Spanish speakers from Spain were tested with different Spanish regional accents from Spain and Latin-America. The results of this experiment replicated the illusory truth effect by showing that repeated statements were judged more credible than new (non-repeated) statements. However, this effect was not modulated by accent. The authors argued that both types of regional accents (i.e., Spanish and Latin-American) were highly intelligible for the Spanish participants they tested, which might reduce the space for finding an interaction of accent on credibility.^1^ There was however another aspect of Frances and colleagues’ design that might have reduced the possibility of finding an accent modulation in their study. Indeed, the authors used a mixed presentation of the statements: while in the first encoding phase the statements were aurally presented, in the critical judgment phase the statements were presented in written modality. The written modality can reduce the impact of fluency since both types of statements in the judgment phase (i.e., those ascribed to the two regional accents) would not differ in terms of fluency processing. In our current study, we used an aural presentation of the statements in both the encoding phase and the critical judgment phase.

Another feature of our study is that we considered the influence of several variables. One was the role of adaptation to foreign accents in judgments of credibility (Clarke & Garrett, 2004; Rovetti et al., 2023; Boduch-Grabka & Lev-Ari, 2021). As mentioned above, several studies suggested that the difficulty of processing foreign-accented speech is reduced with previous exposure to the accent. Exposure involves an adaptation mechanism that would reduce the difference in judgments of credibility between foreign- and native-accented speech. In our study, we aim to mitigate adaptation to better explore the impact of fluency processing on credibility. In doing so, we included a large number of foreign speakers from all over the world. Specifically, 20 foreign speakers originally from 16 countries were selected to record the statements. The inclusion of so many foreign speakers from so many different origins makes it difficult for our participants to adapt to their accents. At the same time, including a wide range of speakers helps minimize the potential impact of variables associated with, for example, accent familiarity (see Derwing & Munro, 1997; Wetzel, Zufferey & Gygax, 2021) and prestige (Giles, 1970). Given that these factors often hinge on individual differences that are challenging to regulate, our preference was to incorporate various accents. To the best of our knowledge, this is the biggest number of foreign speakers from different origins ever tested in message credibility. In addition, to further control for any possible role of adaptation on credibility, the data were analysed with regression models performed at the single trial level. This provides us with a fine-grained statistical approach, in which the properties of each participant and statements are considered, allowing us to test the impact of statement order at a participant level.

Furthermore, it has been shown that listeners extract personality judgments from unknown speakers from the utterance of a single word (e.g., “hello”; McAleer, Todorov & Belin, 2014; Baus et al., 2019), suggesting that the voice is a powerful cue that shapes the personal impressions of others. Moreover, studies in the literature have shown that accent negatively affect the evaluation of speakers on different social variables (Foucart et al., 2020). As a last control measure, we asked a new group of participants to rate the extent to which 11 social traits characterized the speakers of our experiment. Based on past research on language attitudes (Dragojevic & Giles, 2016), we chose five traits related to status (i.e., brilliant, educated, smart, competent, successful) and six related to solidarity (i.e., friendly, nice, pleasant, honest, sociable, trustworthy). In further statistical analyses, we measured the influence of those traits in truth-judgments.

## The present study

The purpose of the present study was to test the impact of foreign-accented speech on message credibility. The illusory truth paradigm was adopted, in which two groups of native Italian speakers were exposed to audio statements in Italian, presented by either native or foreign speakers of the language. We recorded sentences from twenty native and twenty foreign-accented speakers. The foreign speakers came from several parts of the world and did not have Italian as their native language. Following research on the illusory true effect (Dechêne et al., 2010), statements consisted of trivia facts about general world knowledge that were mostly unknown to the Italian population (Lorenzoni et al., 2022b). The experiment was divided into three phases. In the first encoding phase, participants listened to statements and had to decide whether they found the sentences interesting or uninteresting. After a brief math-distractor task lasting a few minutes, participants proceeded to the critical truth-judgment test phase. During this phase, the statements presented in the encoding phase (i.e., repeated condition) were presented alongside new statements (i.e., new condition) in random order. In this test phase, participants had to judge the truthfulness of the statements. Two versions of the experiments were created, one featuring statements recorded by native speakers and the other with statements recorded by foreign speakers. Participants were randomly assigned to one of the two versions. The same statements were used in both versions, and the procedures were identical between them, except for the speakers (native or foreign).

We expected to replicate the illusory truth effect and to observe higher true ratings for repeated statements than for new statements. Additionally, if foreign-accented speech hampers fluency processing, we should observe an interaction of this effect with the accent. Specifically, we anticipate a weaker illusory truth effect in the foreign-accented group compared to the native-accented group. Research in the illusory truth by Hawkins and Hock (1992) suggests a positive correlation between understandability and the magnitude of the effect. Specifically, statements rated as easy to understand produce a larger truth effect than statements rated as more difficult to understand. Their interpretation was that difficult items may not connect, or may connect more weakly, with existing knowledge. According to the referential theory of the illusory truth effect (Unkelbach & Rom, 2017), lower activation of linked memory would reduce the illusory truth effect (see Nadarevic, Plier, Thielmann & Darancò, 2018, for partially congruent evidence). Furthermore, to explore the role of accent independently of the illusory truth effect (i.e., repetition), we compared ratings on new statements only. This comparison is similar to the one conducted in the original study by Lev-Ari and Keysar (2010), in which statements were presented just once. Again, we should observe a decrease in credibility for the new condition statements delivered by foreign-accented speakers compared to those delivered by native-accented speakers.

## Method

### Participants

A ‘snowball’ procedure was used for participant enrolment through social media. We collected data from 60 native Italian speakers (mean age = 24.69, SD = 5.53; 38 females and 22 males). The sample size was fixed to 60 participants based on the recommendation that, in a regression analysis (see the results section), increasing the number of observations by 5–10 per variable is likely to provide at least an acceptable estimation of regression coefficients, standard errors, and confidence intervals (Bentler & Chou, 1987; Bollen, 1989; Hanley, 2016). All participants were required to provide written informed consent, both before and after the experimental session. The test was administered online and anonymously using Labvanced software (Finger et al., 2017). The Research Ethics Committees of the University of Padova approved the experimental procedures (Protocol number: 4404).

### Materials

The experimental set was composed of 80 unknown Italian sentences containing statements about world knowledge facts mostly unknown to Italian population (e.g., ‘Leprosy is caused by a bacterium’). The experimental set was taken from a previous study (Lorenzoni et al., 2022b). The sentences were recorded by 40 speakers (20 females and 20 males). Twenty speakers were native Italian speakers (mean age = 28.95, SD = 9.94; range = 20–55 years), and the other twenty foreign speakers (mean age = 33.25, SD = 13.77; range = 18–61 years). Ten of the Italian speakers were male and the same was true for the foreign speakers. The foreign speakers had a strong foreign-accented speech in Italian and different accents, as they came from various part of the world, including Brazil, China, Colombia, Costa Rica, France, Germany, Hungary, Israel, Japan, Moldova, Iran, Philippines, Romania, Russia, Spain, Ukraine. The eighty sentences were randomly divided into 2 sets of 40 sentences each, and each speaker was asked to record two sentences for each set. In doing so, each speaker contributed with four experimental sentences. The sentences were recorded in a soundproof room and were then edited with Audacity software (v 2.0.3). Recording durations for foreign-accented sentences (mean = 4221 ms; SD = 1279; range = 1881–8870] and Italian sentences [mean = 3349 ms; SD = 856; range = 1660–5538) differed (t(158) = –5.06, p =< .001).

### Procedure

The experiment was conducted as a web-based study. Upon clicking on the invitation link to the study and having accepted the informed consent, participants were randomly assigned to the native-accented or the foreign-accented condition. The experiment consists of three phases, following the standard procedure of the illusory truth paradigm: encoding, math task, truth-judgment test. In the first encoding phase, 40 sentences were aurally presented, and participants had to rate whether they considered the sentence “interesting” (by pressing the A key on the keyboard) or “uninteresting” (by pressing the L key on the keyboard). Trial structure was the following: a fixation cross appeared on the screen for 500 ms, followed by the aurally presentation of the statement. Participants had a maximum of 6 s to respond to the judgment of interest. Each of the 20 speakers in the native and foreign versions contributed with two sentences in the encoding phase. Once the encoding phase was completed, participants performed a distractor-match task. In this task participants were required to decide whether simple arithmetical problems (e.g., 4 × 7 = 28; (5 + 3)/2 = 5) were correct or not by pressing a button on the keyboard. This task lasted 5 minutes and was presented to avoid recency effects. In the third and critical truth-judgment test phase, the same forty familiarization sentences presented in the encoding phase (i.e., repeated condition) were presented randomly together with forty new statements (i.e., new condition). Participants listened the four sentences recorded by each speaker of each version. Trial structure was the same as in the encoding phase with the difference that participants had a maximum of 7 s to judge the veracity of the sentences on a 6-point Likert scale (1- “completely false” and 6- “completely truth”). We adopted a 6-point Likert scale as a frequently used measure to detect truth-judgment differences with unknown statements (Frances et al., 2018; Hanzlíková & Skarnitzl, 2017; Lorenzoni et al., 2022b). Furthermore, the use of an even-point Likert scale prevents participants from selecting the middle option, which typically indicates “I don’t know”.

To ensure that each sentence was presented in both the repeated and new conditions, the experimental statements were randomly divided into two sets of forty each (set A and B), and two lists were created. In list 1, the sentences from set A were presented in the repeated condition, and the sentences from set B were included in the new condition. Conversely, for list 2, sentences from set A were presented in the new condition and sentences from set B were included in the repeated condition. To ensure that participants paid attention throughout the course of the study, we added 4 catch-trials in the encoding phase and 5 catch-trials in the truth-judgment test phase, in which participants were asked to press a specific number on the keyboard.

After the test phase concluded, participants were asked to evaluate the intelligibility (rated on a 6-point Likert scale from 1- “very difficult to understand” and 6- “extremely clear”) and perceived accent (rated on a 6-point Likert scale from 1- “Italian” and 6- “clearly foreign/not Italian”) of each speaker. They listened to one sentence from each voice they had heard during the encoding phase. The entire experimental session lasted 20 minutes.

### Speaker’s traits ratings

A new group of 109 participants (mean age = 31.07, SD = 11.28, 82 females and 27 males) from the same population who did not participate in the main experiment were recruited. For the sake of clarity, we refer to these participants as evaluators. The evaluators were presented with one new sentence from each of the 40 speakers and required to rate one personal trait on a 7-point Likert scale (1 = not at all, 7 = very). In doing so, the evaluators listened to the same sentence said by each speaker, for 40 times. The order of speaker was randomized across evaluators. Five traits were related to status (i.e., brilliant, educated, smart, competent, successful), and six others were related to solidarity (i.e., friendly, funny, pleasant, honest, sociable, trustworthy). The number of evaluators that rated each of these traits was 10, 10, 9, 10, 10, 10, 10, 11, 10, 10 and 9, respectively. As a further measure of control, all the evaluators had to rate the intelligibility and perceived accent of each speaker at the end of the experiment using a Likert-Scale: 1- “very difficult to understand” and 6- “extremely clear” for intelligibility, and 1- “Italian” and 6- “clearly foreign/not Italian” for perceived accent.

### Analysis

Analyses were performed on the responses in the truth-judgment test phase using R software (RC Team, 2018). We employed ordinal logistic regression in the form of a cumulative link mixed model (Christensen, 2015), as implemented by the function *clmm* of the Ordinal package (Christensen, 2018). Different statistical models were compared. We first explored the *Illusory truth effect* by considering the influence of Repetition (Repeated vs New), Accent (Native vs Foreign), Order of presentation, and the interaction between them as fixed effects in the truth-judgment ratings. Participants and Items were included in the models as random effects. In a second-level analysis, *Speaker’s traits influence*, we explored the influence of speaker’s voices in terms of the status and solidarity social traits on the truth-judgment ratings. We also considered the influence of intelligibility and perceived accent traits. Those factors for which there was a significant difference between native and foreign-accented speakers were added to the statistical model of the Illusory truth effect. Finally, in a third-level of analysis, we explored the influence of accent in the new sentences, *New condition analysis*. As detailed above, this analysis allowed us to test the impact of foreign-accented in a manner similar to the study of Lev-Ari and Keysar (2010).

For model comparisons, the fits of the models were compared using the Akaike information criterion (AIC; Akaike, 1987). AIC compares the models at once and provides information about a model’s relative evidence, indicating that the model with the lowest AIC fits best (Wagenmakers & Farrell, 2004). All data is available in the following OSF repository (https://osf.io/mcbjw/).

## Results

From the 62 participants who performed the experiment, we excluded one participant who took more than two hours to complete the experiment and one participant who failed to respond to 4 out of 6 the catch-trials. Data from 60 participants were considered in the analysis. Missing trials (where the participant did not respond) were excluded from the analysis (3.40%).

### Illusory truth effect

**Figure 1:**
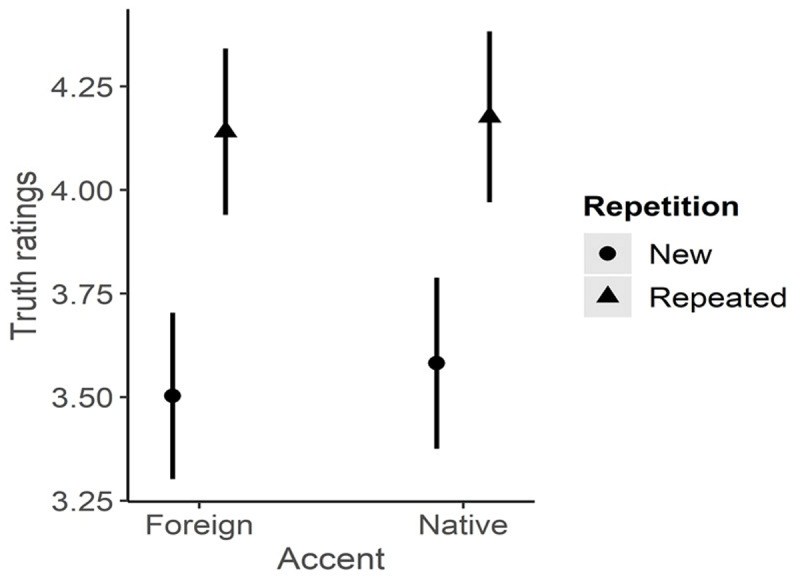
Plot effects of the interaction model on truth ratings. An illusory truth effect of the same amount was observed in both Foreign and Native-accented groups.

In the first type of analysis, the comparison between the models revealed that the best model included the interaction of the three factors (see Appendix A for model comparison). A main effect of the Repetition (Estimate = 1.25; S.E. = .16; z = 7.93; p =< .001; 95% C.I. [2.56 – 4.74]) and an interaction between Repetition and Order (Estimate = –.008; S.E. = .003; z = –2.34; p = .02; 95% C.I. [.99–1]) were observed. Truthfulness ratings were higher in the repeated condition than in the new condition by the same amount for both the native and foreign-accented conditions (see Table 1 and Figure 1). The interaction between Repetition and Order showed that ratings of truthfulness decreased as the order of presentation increased in the repeated condition only. We interpreted this result as a *recency* effect; increasing the temporal delay between the two presentations (i.e., in the encoding and test phases) may reduce the fluency process and, thus, the truthfulness ratings. Since the effect of Order did not interact with Accent and is beyond the scope of the current research, we will not discuss this effect further.

**Table 1:** Truth ratings (scale 1–6) for new and repeated statements. Standard deviations in parentheses. Truth effects are calculated as the difference between repeated and new statements ratings. Accent effects are calculated as the difference between native-accent and foreign-accent ratings.


ILLUSORY TRUTH	NEW	REPEATED	TRUTH EFFECT

Native accent	3.58 (1.26)	4.17 (1.38)	.59

Foreign accent	3.50 (1.40)	4.15 (1.53)	.65

*Accent effect*	.08	.02	–.06


### Speaker’s traits influence

Evaluators judged native and foreign speakers differently on 8 out of the 11 social traits. Evaluators also assessed native and foreign speakers differently in terms of intelligibility and perceived accent. Participants in the main experiment did not judge native and foreign speakers differently in terms of intelligibility, but they perceived foreign-accented speakers as more accented than native-accented. See Table 2 for statistics.

**Table 2:** Results from the 109 evaluators who rated 11 social traits, intelligibility, and perceived accent of the voices used in the main experiment. The asterisk (*) indicates the ratings on intelligibility and perceived accent of the participants who completed the main experiment.


	NATIVE	FOREIGN	STATISTICS (COMPARISON)

Brilliant	4.82 (1.24)	4.45 (.99)	Estimate = .80; S.E. = .19; **z = 4.11**

Educate	5.62 (1.14)	5.74 (1.04)	Estimate = –.30; S.E. = .19; z = –1.57

Smart	4.63 (1.03)	4.45 (.88)	Estimate = .52; S.E. = .21; **z = 2.43**

Competent	5.2 (1.47)	4.69 (1.53)	Estimate = 1.62; S.E. = .24; **z = 6.61**

Successful	4.5 (1.38)	3.86 (1.08)	Estimate = 1.16; S.E. = .19; **z = 6.06**

Friendly	4.68 (1.62)	4.55 (1.42)	Estimate = .24; S.E. = .19; z = 1.28

Funny	4.38 (1.17)	4.09 (1.31)	Estimate = .39; S.E. = .18; **z = –2.13**

Pleasant	5.16 (1.23)	4.89 (1.22)	Estimate = .52; S.E. = .18; **z = 2.89**

Honest	4.72 (1.74)	4.14 (1.77)	Estimate = .73; S.E. = .18; **z = 3.96**

Sociable	4.39 (1.22)	4.37 (1.11)	Estimate = .13; S.E. = .18; z = .69

Trustworthy	5.54 (1.18)	5.39 (.99)	Estimate = .49; S.E. = .20; **z = 2.42**

Intelligibility	6.34 (.97)	5.68 (1.25)	Estimate = 1.93; S.E. = .07; **z = 26.84**

Perceived accent	1.64 (1.38)	5.96 (1.54)	Estimate = –4.66; S.E. = .09; **z = –48.48**

Intelligibility*	5.30 (1.02)	5.07 (1.19)	Estimate = .62; S.E. = .36; z = 1.73

Perceived accent*	4.16 (1.72)	1.59 (1.04)	Estimate = –3.74; S.E. = .32; **z = –11.8**


A total of 11 traits were judged differently between native and foreign speakers (as shown in Table 2 with statistic values in bold). These traits were added to the best model of the first analysis to explore whether they modulated the observed effects in the main experiment. Additive models were tested because the interactive models failed to converge, even when removing the Participants factor from the random structure of the models, as suggested by Bates, Kliegl, Vasishth and Baayen (2015). The addition of none of these traits improved the model fit (see Appendix B for the reported statistics of each of the traits).

### New condition analysis

The results of *clmm* did not report differences between truth judgments in the foreign-accent speaker condition and native-accent speaker condition (Estimate = .15; S.E. = .17; z = .85; p = .39).

## General discussion

The purpose of this study was to investigate whether message credibility is influenced by the accent in which the statement is delivered. We employed the illusory truth paradigm to explore whether the credibility (i.e., truth ratings) of unknown statements differ as a function of the accent, either foreign or native. Our findings revealed that truthfulness ratings were higher for repeated sentences compared to new sentences, replicating previous research on the illusory truth effect (e.g., DiFonzo et al., 2016; Hassan & Barber, 2021). Importantly, our results showed that the illusory truth effect was not modulated by the factor accented-speech, meaning that the increase in credibility was observed in repeated sentences in both the accented-speech versions of the task.

Critically, the experimental design we adopted controlled for several variables to better characterize the influence of accented-speech on message credibility. First, different social traits were extracted from the speakers’ voices by a group of evaluators. Evaluators rated eight of these traits lower when attributed to the foreign speakers as compared to the native speakers, indicating a negative bias versus foreigners and replicating previous research (Dewaele & McCloskey, 2015; Fraser & Kelly, 2012; Fuertes et al., 2012; Gluszek & Dovidio, 2010). However, none of these traits seem to have had an influence on the truthfulness judgments of the main experimental task. That is, the traits tied to the speaker voices did not modulate the main effect on credibility judgments.

As an additional control measure, both the participants in the main experiment and the evaluators assessed that foreign speakers were perceived to have more pronounced foreign accent compared to native speakers. This suggests that our primary manipulation effectively distinguished between foreign and native speech. Furthermore, although the evaluators did report the foreign speakers as less intelligible than the native speakers, this difference was not observed among participants in the main experiment. It is worth noting that the participants in the main experiment had greater exposure to the speaker voices. While the evaluators were exposed to just one sentence, participants in the main experiment heard four sentences from each speaker. This increased exposure likely contributed to the participants’ improved intelligibility of the voices, suggesting that adaptation to accents is a rapid process that occurs after just a few exposures to the voices (Boduch-Grabka & Lev-Ari, 2021; Clarke & Garrett, 2004; Rovetti et al., 2023). In relation to this, Boduch-Grabka and Lev-Ari proposed that adaptation might be a crucial factor in the influence of accents on message credibility. Specifically, the more exposure people have to a specific accent, the higher the probability of adapting to it. According to the ‘easier to process-easier to believe’ hypothesis, increased adaptation would reduce the likelihood of observing differences in truth ratings between native-accented and foreign-accented speakers (Boduch-Grabka & Lev-Ari, 2021). One might argue that our results could be interpreted in the same way. However, we find this interpretation unlikely because we used a large number of speakers (i.e., 40 speakers), each of whom produced four sentences. In contrast, in their original study, Lev-Ari and Keysar’s (2010) used only three speakers who delivered fifteen statements each, and they still observed a reliable interaction between truth judgments and accent.

It is important to note that the repetition of sentences can enhance the intelligibility of the voices, not because the speakers become more intelligible overall, but due to the repetition of the message itself. In other words, it is the repetition of the message that increases the intelligibility, rather than a process of adaptation to the voice. If that were the case, one might expect a ceiling effect in the repeated sentences (i.e. the repetition condition); with no differences in truth ratings between the native and foreign speakers. However, we can rule out this possibility since differences in truth ratings among foreign and native accented statements were not even observed in the new condition, where sentences were presented just once.

The findings we reported here have relevant theoretical and empirical implications. As detailed in the introduction, two factors could be responsible for the bias toward foreign-accented speakers in credibility judgments. One factor is reflected in the processing fluency hypothesis: foreign-accented speech is harder to understand, this implies a reduction of cognitive resources devoted to message processing, and this affects in turn negatively the credibility. A second possible factor is the automatic categorization of the speaker as a foreign individual that activates negative stereotypes. In our experiment we used a between-participant design, so participants were assigned either to the foreign-accent or to the native-accent version of the task. We adopted this strategy to diminish the formation of social categories and, consequently, minimize the activation of stereotypes. This approach allows for a more direct examination of the processing fluency hypothesis. Undoubtedly, the social categorization of the foreign speakers as an out-group may occur automatically in the group of participants exposed to foreign speakers. However, it is less probable the activation of the in-group (native) social category in those participants exposed to the native speakers (Tajfel et al., 1971). Even using a between-participant design, our results did not show a main effect of group nor an interaction with the effect of repetition. Under the assumption that foreign-accented speech is harder to process, and the assumption that between-participant design reduces the impact of social categorization, our findings cannot be accounted for the processing fluency hypothesis.

This research offers a conceptual replication of the pioneer study by Lev-Ari and Keysar (2010), with the aim of replicating the observation that foreign-accented speech diminishes credibility. However, our findings did not support this observation. It is worth noting that our results are in agreement with other research that also did not show differences in credibility between foreign-accented and native-accented speech (Souza & Markman, 2013; Stocker, 2017; see for an example with regional accent Frances et al., 2018). At the same time, other studies have partially confirmed the pattern of Lev-Ari and Keysar (2010), see Hanzlíková & Skarnitzl (2017) and Podlipský et al. (2016). Thus, further research is needed to explore the boundary conditions under which this effect occurs (see for relevant discussion, Foucart et al., 2020).

Recent research suggests that the mere categorization of a speaker as either a foreigner or a native, beyond fluency considerations, influences message credibility. In a study conducted by Lorenzoni and colleagues (2022b), participants were presented with biographical descriptions of two speakers: one characterized as having a native accent and the other as having a foreign accent. Subsequently, participants assessed the truthfulness of written sentences attributed to either the native or the foreign speaker. The sentences consisted of trivial facts mostly unknown to the participants, similar to those used in this research. A noteworthy aspect of Lorenzoni and colleagues’ design is that the written presentation of the statements maintained equal processing fluency between native and foreign speakers. Put simply, sentences associated with both the foreign and native speakers were equally easy or difficult to process. Notably, trustworthiness ratings increased when participants believed that statements were linked to the foreign speaker -a reverse pattern compared to the findings reported by Lev-Ari and Keysar (2010) with accented-speech statements. Lorenzoni and colleagues explained their results by suggesting that individuals tend to be more lenient towards foreign speakers when evaluating unknown statements. In situations where participants lack sufficient information to assess the truth of a statement, they may base their judgment on the perceived knowledge of the speaker. While the knowledge attributed to a native speaker aligns closely with the participant’s own knowledge, participants may attribute a range of knowledge to foreign speakers that differs from their own and tend to trust more foreign speaker knowledge. In sum, participants were more leniency towards foreigners. It is important to note that greater leniency towards foreign speakers compared to native speaker has been also proposed concerning pragmatic and linguistic competence (Fairchild & Papafragou, 2018; Fairchild, Mathis & Papafragou, 2020; Lorenzoni et al., 2022b).

In line with this argument, Roessel, Schoel, and Stahlberg (2020) have recently hypothesized that native listeners might also be aware of the stigma associated with non-native speakers, making them less willing to express opinions that could contribute to that stigma. According to these authors, in certain social situations, people may be tolerant and unbiased toward foreign-accented speakers. Consequently, we cannot exclude the possibility that two forces are interacting in the reported results: a lenience towards foreign speakers contributing to increased truth judgments, while simultaneously, less fluent processing diminishes these judgments. Future research can explore this hypothesis.

In summary, studies that have explored the role of accented-speech on trivia unknown statement judgments have yielded apparently inconsistent results. These studies have also identified some of the factors that affect message credibility for foreigners, such as fluency processing, social categorization, stereotype attribution, and lenience. These factors might not be mutually exclusive. We conclude that in order to offer a precise description of the influence of foreign-accented speech on message credibility, research needs to take into account several social and cognitive factors.

## Data Accessibility Statement

All data and script for analysis are available under the following OSF repository: https://osf.io/mcbjw/.

## Footnotes

In addition, while foreign accents can be attributed to a less linguistic competence because the speaker is a non-native speaker, this is not the case with regional accents where the speaker is speaking in their native language.

## Appendix

**Appendix A:** Statistics of model selection for illusory truth effect analysis. The model which best explained the data is m4, which included the interaction Repetition × Accent × Order as fixed effects.

MODEL	EFFECTS	DF	AIC	BIC	p-VALUE

m0	~1 + R.E.		15374	15418.94	

m1	Repetition + R.E.	1	15092	15143.37	<.001

m2	Repetition + Accent + R.E.	1	15094	15151.75	.79

m3	Repetition + Accent + Order + R.E.	1	15183	15247.29	1.00

**m4**	**Repetition * Accent * Order** + R.E.	**4**	**15091**	**15181.39**	**<.001**

*Note*. R.E. refers to Participant and Item, which were added as random effects in the model. DF = Degrees of Freedom. AIC = Akaike information criterion; BIC = Bayesian information criterion.

**Appendix B:** Reported statistics speaker’s traits influence to the model of interest (see main text for details).

TRAIT	STATISTIC

Brilliant	Estimate = –.001; S.E. = .06; z = .61; p = .98

Smart	Estimate = .03; S.E. = .11; z = .30; p = .76

Competent	Estimate = .08; S.E. = .10; z = .79; p = .43

Success	Estimate = .003; S.E. = .07; z = .04; p = .96

Funny	Estimate = .001; S.E. = .06; z = .02; p = .98

Pleasant	Estimate = .04; S.E. = .07; z = .61; p = .53

Honest	Estimate = .11; S.E. = .09; z = 1.22; p = .22

Trustworthy	Estimate = .007; S.E. = .09; z = .07; p = .94

Perceived accent	Estimate = –.02; S.E. = .07; z = –.24; p = .80

Intelligibility	Estimate = .23; S.E. = .15; z = 1.52; p = .12

Perceived accent*	Estimate = –.009; S.E. = .04; z = –.20; p = .83

*Note*. *Indicates rate on perceived accent of the participants who completed the main experiment.
